# Supplementary material for: Cold induces brain region-selective cell activity-dependent lipid metabolism
Source: bioRxiv. 2024 Sep 12:2024.04.15.589506. Originally published 2024 Apr 17. Preprint. [Version 2] doi: 10.1101/2024.04.15.589506 (PMC11042364; doi:10.1101/2024.04.15.589506)
Supplement: Supplement 1 [file media-1.pdf]

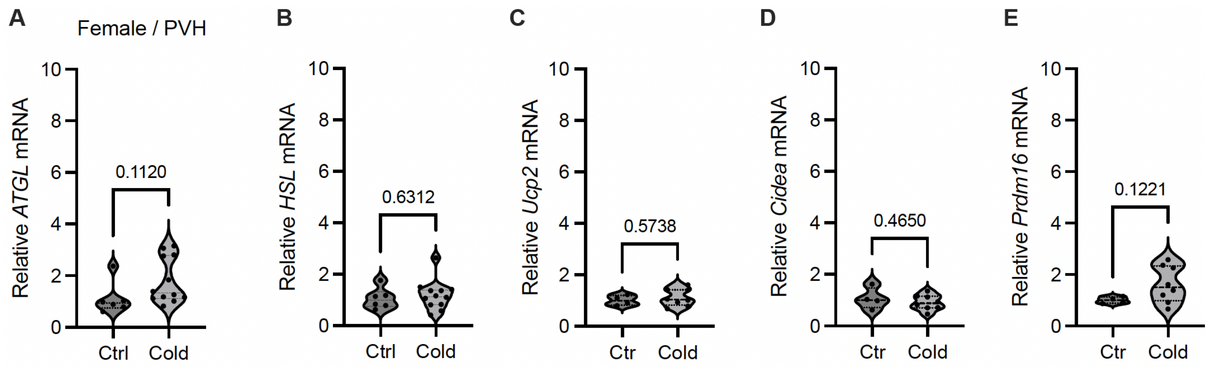

Supplementary Figure 1. Cold did not affect gene markers in PVH in female mice. Micro-punches of PVH were made from female mice exposed to a cold chamber for 4-6 h for RT-qPCR of the gene markers of lipolysis and thermogenesis. Group data of the lipolytic marker *ATGL* (A) and *HSL* (B) as well as thermogenic marker *Ucp2* (C), *Cidea* (D) and *Prdm16* (E) in the PVH. Data represent mean ± s.e.m. Student *t* tests were performed. Each dot represents one animal in each group of all the panels.

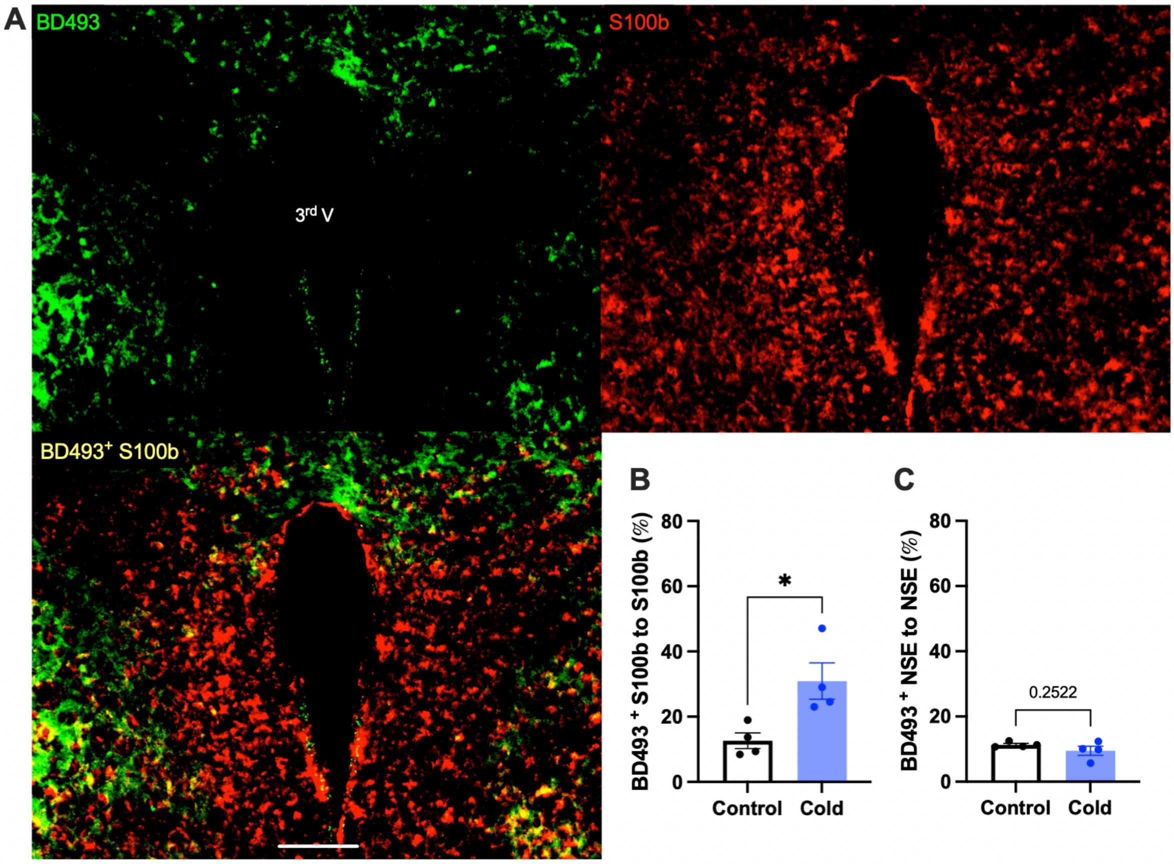

Supplementary Figure 2. Cold increases LD accumulation in astrocytes but not neurons. Control or cold (4- 6)-challenged mouse brains were sectioned. BD493, S100b, and NSE were respectively co-stained using relevant antibodies. (A) Representative images of BD493 (green), S100b (red), and BD493/S100b overlay (yellow) signals in PVH sections from cold-challenged mouse. (B) Group data of relative BD493/S100b overlay signals to total S100b signals in control and cold-challenged mice (n = 4 each group). (C) Group data of relative BD493/NSE overlay signals to total NSE signals in control and cold-challenged mice (n = 4 each group). Data represent mean ± s.e.m. Student *t* tests were performed. $*p<0.05$. Scale bar, 100 μm for (A). 3rd V, third ventricle.

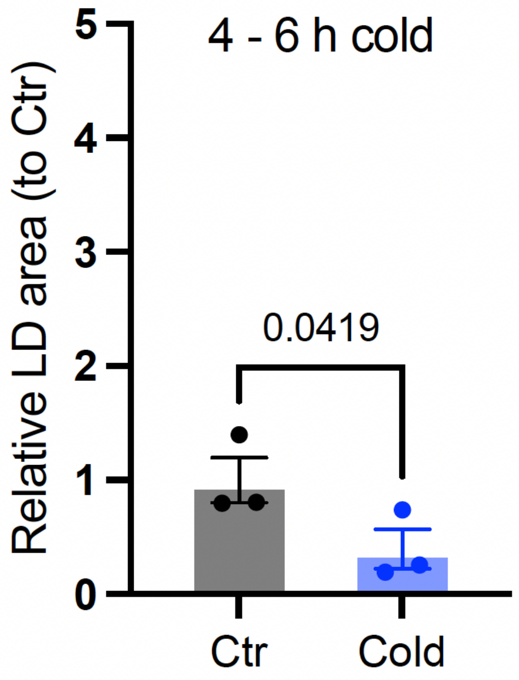

Supplementary Figure 3. Group data of relative BD493-labelled area in PVH in control or cold-challenged (4 ~ 6 h) mice (n=3 each group). Data represent mean ± s.e.m. Student *t* tests were performed.

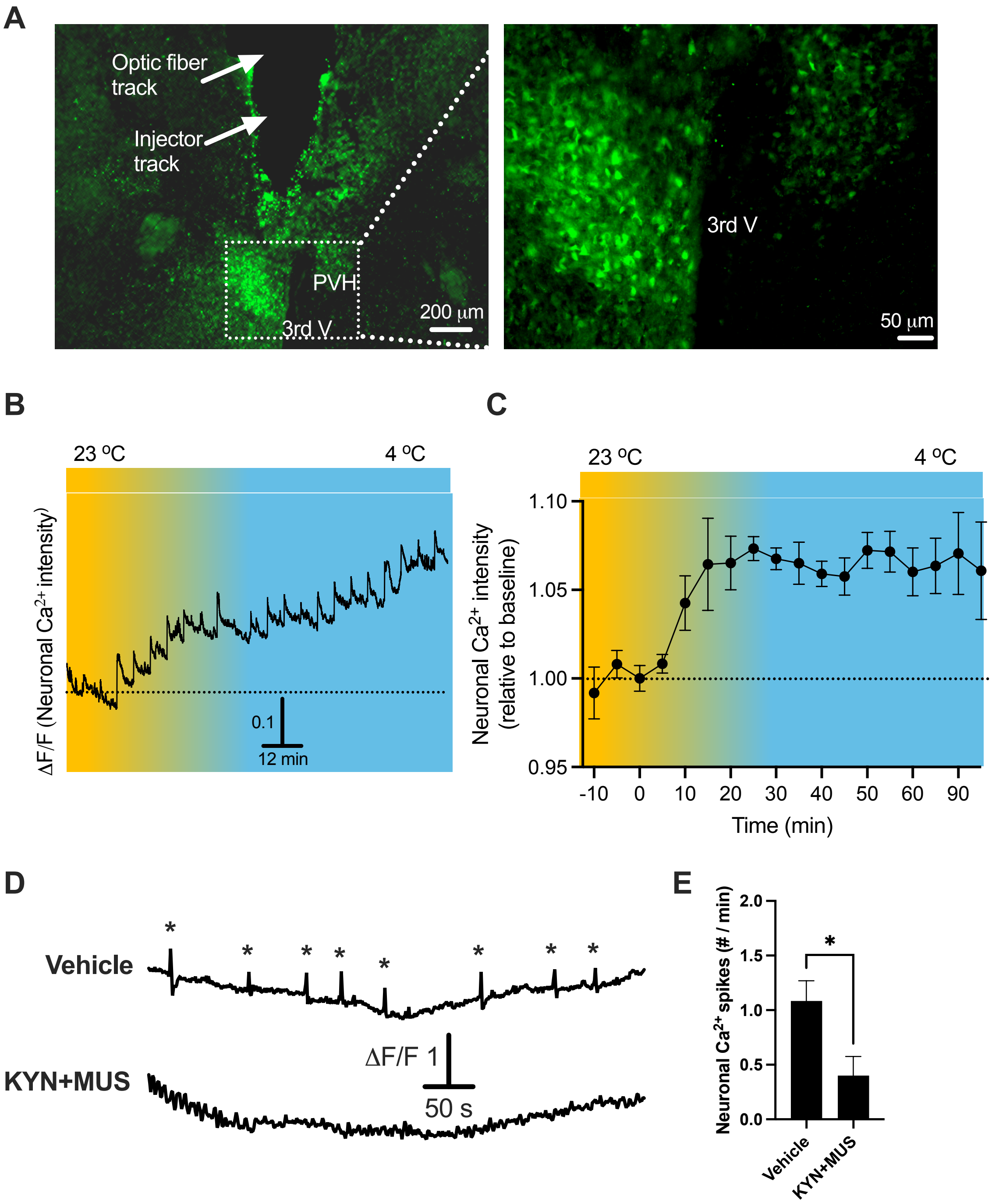

Supplementary Figure 4. Neuronal inhibition by KYN+MUS. (A) Representative images of GCaMP$_{6f}$ expressions in PVH neurons and cannula track. (B) Representative trace of neuronal GCaMP$_{6f}$ signals recorded in a time lapse manner in a cold chamber. (C) Group data of (B, n=3). (D) Representative traces of neuronal GCaMP$_{6f}$ signals recorded from vehicle (top) and KYN+MUS (bottom)-injected mice, and stars indicate Ca^{2+} spikes. (E) Group data of relative GCaMP$_{6f}$-labelled Ca^{2+} spikes in PVH neurons in vehicle or KYN+MUS-injected mice (n=3 each group). Data represent mean ± s.e.m. Student *t* tests were performed. *p<0.05 for (E).
